# [μ-1,1′-Bis(diphenyl­phosphino)ferrocene]bis­{[(*Z*)-*O*-ethyl *N*-phenyl­thio­carbamato-κ*S*]gold(I)} dichloro­methane solvate

**DOI:** 10.1107/S160053681001562X

**Published:** 2010-05-08

**Authors:** Soo Yei Ho, Edward R. T. Tiekink

**Affiliations:** a Department of Chemistry, National University of Singapore, Singapore 117543; b Department of Chemistry, University of Malaya, 50603 Kuala Lumpur, Malaysia

## Abstract

The binuclear title compound, [Au_2_Fe(C_9_H_10_NOS)_2_(C_17_H_14_P)_2_]·CH_2_Cl_2_, which has the Fe atom located on a crystallographic centre of inversion, crystallizes as a 1:1 dichloro­methane solvate, which is disordered about a centre of inversion. There is a small deviation from linearity defined by the *SP* donor set [S1—Au—P1 angle is 175.35 (5) °] which is due to an intra­molecular Au⋯O contact [3.080 (5) Å]. The primary inter­molecular contacts between binuclear mol­ecules are of the type C—H⋯π, and are arranged so as to form columns in the *a*-axis direction in which the disordered solvent mol­ecules reside.

## Related literature

For the structural systematics and luminescence properties of phosphinegold(I) carbonimidothio­ates, see: Ho *et al.* (2006 ▶); Ho & Tiekink (2007 ▶); Kuan *et al.* (2008 ▶). For the synthesis, see: Hall *et al.* (1993 ▶). For related structures, see: Ho & Tiekink (2009 ▶); Tadbuppa & Tiekink (2009 ▶).
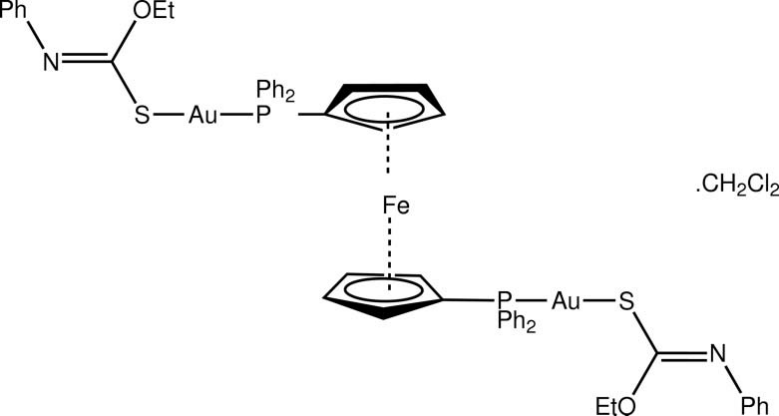


## Experimental

### 

#### Crystal data

[Au_2_Fe(C_9_H_10_NOS)_2_(C_17_H_14_P)_2_]·CH_2_Cl_2_
*M* *_r_* = 1393.69
Triclinic, 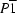

*a* = 8.442 (3) Å
*b* = 12.957 (5) Å
*c* = 13.440 (5) Å
α = 108.045 (8)°
β = 103.177 (8)°
γ = 106.853 (8)°
*V* = 1253.5 (9) Å^3^
*Z* = 1
Mo *K*α radiation
μ = 6.42 mm^−1^
*T* = 223 K
0.49 × 0.04 × 0.04 mm

#### Data collection

Bruker SMART CCD diffractometer
Absorption correction: multi-scan (*SADABS*; Bruker, 2000 ▶) *T*_min_ = 0.577, *T*_max_ = 1
8618 measured reflections
5688 independent reflections
5025 reflections with *I* > 2σ(*I*)
*R*_int_ = 0.030

#### Refinement

*R*[*F* ^2^ > 2σ(*F* ^2^)] = 0.038
*wR*(*F* ^2^) = 0.106
*S* = 1.02
5688 reflections
307 parameters
13 restraints
H-atom parameters constrained
Δρ_max_ = 2.84 e Å^−3^
Δρ_min_ = −1.47 e Å^−3^

### 

Data collection: *SMART* (Bruker, 2000 ▶); cell refinement: *SAINT* (Bruker, 2000 ▶); data reduction: *SHELXTL* (Sheldrick, 2008 ▶); program(s) used to solve structure: *PATTY* in *DIRDIF92* (Beurskens *et al.*, 1992 ▶); program(s) used to refine structure: *SHELXL97* (Sheldrick, 2008 ▶); molecular graphics: *ORTEP-3* (Farrugia, 1997 ▶) and *DIAMOND* (Brandenburg, 2006 ▶); software used to prepare material for publication: *publCIF* (Westrip, 2010 ▶).

## Supplementary Material

Crystal structure: contains datablocks global, I. DOI: 10.1107/S160053681001562X/pk2245sup1.cif

Structure factors: contains datablocks I. DOI: 10.1107/S160053681001562X/pk2245Isup2.hkl

Additional supplementary materials:  crystallographic information; 3D view; checkCIF report

## Figures and Tables

**Table 1:** Selected bond lengths (Å)

Au—P1	2.2562 (15)
Au—S1	2.3029 (16)

**Table 2:** Hydrogen-bond geometry (Å, °) *Cg*1 and *Cg*2 are the centroids of the C2–C7 and C15–C20 rings, respectively.

*D*—H⋯*A*	*D*—H	H⋯*A*	*D*⋯*A*	*D*—H⋯*A*
C9—H9a⋯*Cg*1^i^	0.97	2.75	3.623 (9)	150
C11—H11⋯*Cg*2^ii^	0.94	2.78	3.619 (7)	150

Symmetry codes: (i) 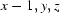; (ii) 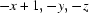.

**Fig. 1.**
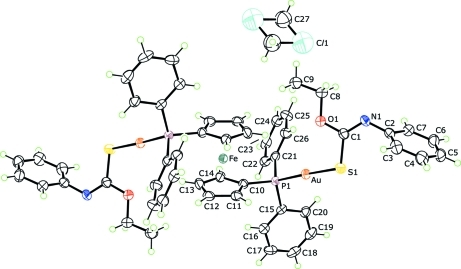
Molecular structure of the dinuclear complex (I) showing atom-labelling scheme and displacement ellipsoids at the 50% probability level. The dinuclear molecule is located about a centre of inversion and unlabelled atoms are related by the symmetry operation -x, -y, -z. Also shown is the solvent dichloromethane molecule which is disordered about a centre of inversion. Unlabelled atoms are related by -x, 1-y, 1-z for this molecule.

**Fig. 2.**
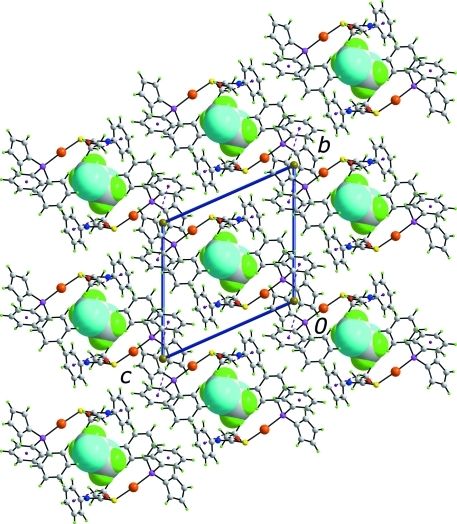
A view in projection down the a axis of the crystal packing in (I) highlighting the interactions between binuclear molecules mediated by C–H···π contacts (purple dashed lines). The binuclear molecules define columns in which reside the disordered dichloromethane molecules, shown in space filling mode. Colour code: Au, orange; Fe, olive green; S, yellow; P, pink; O, red; N, blue; C, grey; and H, green.

## supplementary crystallographic information

### Comment

The dppf (where dppf is the bidentate phosphine, [Ph_2_P(C_5_H_4_)]_2_Fe) derivatives of phosphinegold(I) thiocarbamides, of interest owing to crystal engineering and luminescence studies (Ho *et al.* 2006; Ho & Tiekink, 2007; Kuan *et al.*, 2008), are comparatively rare. Thus, only three examples of dppf{Au[SC(OR)═NR']}_2_ have been described, i.e. R = Me & R' = PhNO_2_-4 (Ho *et al.*, 2006), R = iPr & R' = PhNO_2_-4 (Ho & Tiekink, 2009), and R = iPr & R' = PhMe-4 (Tadbuppa & Tiekink, 2009). In the present report, the crystal structure of the R = Et & R' = H derivative, (I), is described.

The dinuclear molecule has crystallographic symmetry with the Fe atom lying on an inversion centre, Fig. 1. The dinuclear molecule crystallises with a solvent dichloromethane molecule which is disordered about a centre of inversion, Fig. 1. The gold atom exists in the expected linear geometry defined by a *SP* donor set, Table 1, and the deviation from linearity [S1–Au–P1 is 175.35 (5) °] is ascribed to the close approach of the O1 atom, Au···O = 3.080 (5) Å. The anion, with a Z configuration about the C1═N1 bond, shows the expected characteristics. The magnitudes of the C1—S1 and C1═N1 bond distances of 1.755 (6) and 1.277 (8) Å, respectively, confirm that the anion is coordinating as a thiolate ligand. The overall conformation of the molecule is "open" in that the thiocarbamate ligands are lying on either side of the molecule, as found in the structure of the R = iPr & R' = PhMe-4 derivative (Tadbuppa & Tiekink, 2009) but contrasts the situation in each of dppf{Au[SC(OR)═ NC_6_H_4_NO_2_-p]}_2_, for R = Me (Ho *et al.*, 2006) and i-Pr (Ho & Tiekink, 2009), whereby the molecule has a U-shaped conformation allowing for the formation of intramolecular Au···Au interactions.

In the crystal structure of (I), the primary interactions between the dinuclear molecules are of the type C–H···π, Table 1. These are arranged so as to define columns along the *a* direction in which reside the solvent dichloromethane molecules.

### Experimental

Compound (I) was prepared following the standard literature procedure from the reaction of dppf(AuCl)_2_ and EtOC(═S)N(H)Ph in the presence of base (Hall *et al.*, 1993). Crystals were obtained from the slow evaporation of a dichloromethane solution.

### Refinement

The H atoms were geometrically placed (C—H = 0.94-0.98 Å) and refined as riding with *U*_iso_(H) = 1.2-1.5*U*_eq_(C). The maximum and minimum residual electron density peaks of 2.84 and 1.47 e Å^-3^, respectively, were located within the C21–C26 ring (0.95 Å from the C21 atom) and 0.58 Å from the Cl1 atom, respectively. The binuclear molecule co-crystallised with a disordered dichloromethane solvent molecule. This was modelled over a centre of inversion with a full weight chloride and half-weight methylene group. The C and Cl atoms were treated with the ISOR command in SHELXL-97 to impose isotropic character to the anisotropic displacement parameters (Sheldrick, 2008). The following reflections (0,1,0), (0,-1,1) and (0,0,1) were omitted in the final refinement as they were obscured by the beamstop.

### Crystal data

**Table:**

[Au_2_Fe(C_9_H_10_NOS)_2_(C_17_H_14_P)_2_]·CH_2_Cl_2_	*Z* = 1
*M**_r_* = 1393.69	*F*(000) = 678
Triclinic, *P*1	*D*_x_ = 1.846 Mg m^−^^3^
Hall symbol: -P 1	Mo *K*α radiation, λ = 0.71069 Å
*a* = 8.442 (3) Å	Cell parameters from 4599 reflections
*b* = 12.957 (5) Å	θ = 2.6–30.1°
*c* = 13.440 (5) Å	µ = 6.42 mm^−^^1^
α = 108.045 (8)°	*T* = 223 K
β = 103.177 (8)°	Needle, orange
γ = 106.853 (8)°	0.49 × 0.04 × 0.04 mm
*V* = 1253.5 (9) Å^3^	

### Data collection

**Table:**

Bruker SMART CCD diffractometer	5688 independent reflections
Radiation source: fine-focus sealed tube	5025 reflections with *I* > 2σ(*I*)
graphite	*R*_int_ = 0.030
ω scans	θ_max_ = 27.5°, θ_min_ = 2.6°
Absorption correction: multi-scan (*SADABS*; Bruker, 2000)	*h* = −10→10
*T*_min_ = 0.577, *T*_max_ = 1	*k* = −16→11
8618 measured reflections	*l* = −16→17

### Refinement

**Table:**

Refinement on *F*^2^	Primary atom site location: structure-invariant direct methods
Least-squares matrix: full	Secondary atom site location: difference Fourier map
*R*[*F*^2^ > 2σ(*F*^2^)] = 0.038	Hydrogen site location: inferred from neighbouring sites
*wR*(*F*^2^) = 0.106	H-atom parameters constrained
*S* = 1.02	*w* = 1/[σ^2^(*F*_o_^2^) + (0.0684*P*)^2^] where *P* = (*F*_o_^2^ + 2*F*_c_^2^)/3
5688 reflections	(Δ/σ)_max_ = 0.001
307 parameters	Δρ_max_ = 2.84 e Å^−^^3^
13 restraints	Δρ_min_ = −1.47 e Å^−^^3^

### Special details

**Table:**

Geometry. All s.u.'s (except the s.u. in the dihedral angle between two l.s. planes) are estimated using the full covariance matrix. The cell s.u.'s are taken into account individually in the estimation of s.u.'s in distances, angles and torsion angles; correlations between s.u.'s in cell parameters are only used when they are defined by crystal symmetry. An approximate (isotropic) treatment of cell s.u.'s is used for estimating s.u.'s involving l.s. planes.
Refinement. Refinement of *F*^2^ against ALL reflections. The weighted *R*-factor wR and goodness of fit *S* are based on *F*^2^, conventional *R*-factors *R* are based on *F*, with *F* set to zero for negative *F*^2^. The threshold expression of *F*^2^ > 2σ(*F*^2^) is used only for calculating *R*-factors(gt) etc. and is not relevant to the choice of reflections for refinement. *R*-factors based on *F*^2^ are statistically about twice as large as those based on *F*, and *R*- factors based on ALL data will be even larger.

### Fractional atomic coordinates and isotropic or equivalent isotropic displacement parameters (Å^2^)

**Table:**

	*x*	*y*	*z*	*U*_iso_*/*U*_eq_	Occ. (<1)
Au	0.54576 (2)	0.149128 (17)	0.224945 (16)	0.02842 (9)	
Fe	0.0000	0.0000	0.0000	0.0234 (2)	
S1	0.6884 (2)	0.11576 (15)	0.37230 (12)	0.0387 (3)	
P1	0.42701 (17)	0.19357 (11)	0.08358 (11)	0.0240 (3)	
O1	0.4392 (5)	0.1685 (4)	0.4324 (3)	0.0397 (10)	
N1	0.6859 (6)	0.2200 (5)	0.5809 (4)	0.0368 (11)	
C1	0.6054 (8)	0.1739 (5)	0.4754 (5)	0.0317 (11)	
C2	0.8589 (8)	0.2308 (5)	0.6274 (5)	0.0344 (12)	
C3	0.8983 (9)	0.1323 (6)	0.6232 (6)	0.0422 (14)	
H3	0.8092	0.0561	0.5840	0.051*	
C4	1.0693 (9)	0.1475 (6)	0.6769 (6)	0.0439 (14)	
H4	1.0952	0.0810	0.6739	0.053*	
C5	1.2007 (9)	0.2566 (6)	0.7341 (5)	0.0434 (15)	
H5	1.3163	0.2649	0.7690	0.052*	
C6	1.1636 (8)	0.3563 (6)	0.7409 (6)	0.0440 (15)	
H6	1.2529	0.4323	0.7808	0.053*	
C7	0.9909 (8)	0.3404 (6)	0.6873 (5)	0.0394 (13)	
H7	0.9642	0.4070	0.6924	0.047*	
C8	0.3614 (8)	0.2141 (7)	0.5128 (6)	0.0466 (16)	
H8A	0.4412	0.2944	0.5658	0.056*	
H8B	0.3408	0.1646	0.5546	0.056*	
C9	0.1920 (10)	0.2139 (8)	0.4508 (6)	0.0551 (19)	
H9A	0.1370	0.2436	0.5031	0.083*	
H9B	0.1142	0.1342	0.3983	0.083*	
H9C	0.2139	0.2640	0.4106	0.083*	
C10	0.2261 (7)	0.0814 (4)	−0.0219 (4)	0.0255 (10)	
C11	0.1892 (7)	−0.0416 (5)	−0.0552 (5)	0.0312 (11)	
H11	0.2615	−0.0753	−0.0247	0.037*	
C12	0.0209 (8)	−0.1038 (5)	−0.1443 (5)	0.0377 (14)	
H12	−0.0363	−0.1860	−0.1833	0.045*	
C13	−0.0434 (8)	−0.0202 (5)	−0.1629 (5)	0.0372 (13)	
H13	−0.1520	−0.0377	−0.2161	0.045*	
C14	0.0802 (7)	0.0937 (5)	−0.0894 (5)	0.0304 (11)	
H14	0.0693	0.1650	−0.0853	0.036*	
C15	0.5783 (7)	0.2270 (4)	0.0097 (5)	0.0268 (10)	
C16	0.5327 (8)	0.1678 (5)	−0.1046 (5)	0.0320 (11)	
H16	0.4185	0.1101	−0.1479	0.038*	
C17	0.6581 (9)	0.1949 (6)	−0.1545 (5)	0.0395 (13)	
H17	0.6282	0.1551	−0.2319	0.047*	
C18	0.8269 (8)	0.2804 (6)	−0.0905 (6)	0.0426 (15)	
H18	0.9102	0.2990	−0.1247	0.051*	
C19	0.8716 (8)	0.3371 (6)	0.0215 (6)	0.0425 (14)	
H19	0.9861	0.3945	0.0643	0.051*	
C20	0.7489 (7)	0.3110 (5)	0.0738 (5)	0.0335 (12)	
H20	0.7812	0.3498	0.1515	0.040*	
C21	0.3847 (7)	0.3269 (5)	0.1315 (5)	0.0294 (11)	
C22	0.3700 (7)	0.3903 (5)	0.0660 (5)	0.0321 (11)	
H22	0.3774	0.3630	−0.0056	0.038*	
C23	0.3444 (8)	0.4938 (5)	0.1063 (6)	0.0372 (13)	
H23	0.3335	0.5363	0.0615	0.045*	
C24	0.3348 (8)	0.5355 (5)	0.2118 (6)	0.0379 (13)	
H24	0.3199	0.6068	0.2393	0.046*	
C25	0.3471 (9)	0.4717 (6)	0.2762 (6)	0.0454 (15)	
H25	0.3379	0.4988	0.3473	0.055*	
C26	0.3728 (8)	0.3689 (5)	0.2371 (5)	0.0353 (12)	
H26	0.3824	0.3265	0.2821	0.042*	
Cl1	0.1902 (6)	0.5357 (4)	0.5501 (4)	0.1365 (14)	
C27	0.0112 (19)	0.5715 (19)	0.548 (2)	0.085 (6)	0.50
H27A	0.0327	0.6434	0.5342	0.103*	0.50
H27B	0.0018	0.5900	0.6223	0.103*	0.50

### Atomic displacement parameters (Å^2^)

**Table:**

	*U*^11^	*U*^22^	*U*^33^	*U*^12^	*U*^13^	*U*^23^
Au	0.02700 (12)	0.03400 (13)	0.02496 (13)	0.01353 (9)	0.00771 (8)	0.01222 (9)
Fe	0.0212 (4)	0.0246 (5)	0.0227 (5)	0.0075 (4)	0.0068 (4)	0.0093 (4)
S1	0.0421 (8)	0.0566 (9)	0.0265 (7)	0.0317 (7)	0.0117 (6)	0.0171 (6)
P1	0.0230 (6)	0.0252 (6)	0.0242 (6)	0.0100 (5)	0.0086 (5)	0.0098 (5)
O1	0.031 (2)	0.054 (3)	0.028 (2)	0.0181 (19)	0.0081 (17)	0.0088 (19)
N1	0.031 (2)	0.049 (3)	0.021 (2)	0.010 (2)	0.0066 (19)	0.009 (2)
C1	0.032 (3)	0.037 (3)	0.026 (3)	0.015 (2)	0.009 (2)	0.013 (2)
C2	0.033 (3)	0.049 (3)	0.023 (3)	0.016 (2)	0.011 (2)	0.016 (2)
C3	0.041 (3)	0.040 (3)	0.040 (3)	0.009 (3)	0.010 (3)	0.019 (3)
C4	0.051 (4)	0.048 (4)	0.042 (4)	0.025 (3)	0.013 (3)	0.027 (3)
C5	0.035 (3)	0.064 (4)	0.033 (3)	0.021 (3)	0.009 (3)	0.023 (3)
C6	0.030 (3)	0.051 (4)	0.038 (3)	0.007 (3)	0.007 (3)	0.015 (3)
C7	0.031 (3)	0.047 (3)	0.038 (3)	0.015 (3)	0.012 (3)	0.014 (3)
C8	0.037 (3)	0.061 (4)	0.036 (3)	0.022 (3)	0.014 (3)	0.007 (3)
C9	0.052 (4)	0.083 (5)	0.037 (4)	0.042 (4)	0.016 (3)	0.018 (4)
C10	0.026 (2)	0.026 (2)	0.025 (2)	0.0100 (19)	0.008 (2)	0.0101 (19)
C11	0.034 (3)	0.027 (3)	0.034 (3)	0.013 (2)	0.015 (2)	0.011 (2)
C12	0.033 (3)	0.032 (3)	0.035 (3)	0.003 (2)	0.018 (3)	0.002 (2)
C13	0.032 (3)	0.046 (3)	0.023 (3)	0.007 (2)	0.006 (2)	0.011 (2)
C14	0.026 (2)	0.041 (3)	0.027 (3)	0.012 (2)	0.010 (2)	0.019 (2)
C15	0.024 (2)	0.028 (2)	0.035 (3)	0.012 (2)	0.013 (2)	0.017 (2)
C16	0.032 (3)	0.033 (3)	0.034 (3)	0.013 (2)	0.012 (2)	0.015 (2)
C17	0.049 (3)	0.049 (3)	0.037 (3)	0.027 (3)	0.027 (3)	0.022 (3)
C18	0.038 (3)	0.053 (4)	0.065 (4)	0.028 (3)	0.033 (3)	0.040 (3)
C19	0.027 (3)	0.040 (3)	0.061 (4)	0.011 (2)	0.016 (3)	0.022 (3)
C20	0.027 (3)	0.032 (3)	0.038 (3)	0.008 (2)	0.010 (2)	0.013 (2)
C21	0.020 (2)	0.028 (2)	0.035 (3)	0.0059 (19)	0.008 (2)	0.010 (2)
C22	0.030 (3)	0.027 (3)	0.038 (3)	0.011 (2)	0.013 (2)	0.011 (2)
C23	0.028 (3)	0.035 (3)	0.048 (4)	0.012 (2)	0.011 (3)	0.019 (3)
C24	0.034 (3)	0.026 (3)	0.044 (3)	0.010 (2)	0.009 (3)	0.005 (2)
C25	0.048 (4)	0.047 (4)	0.032 (3)	0.019 (3)	0.016 (3)	0.003 (3)
C26	0.038 (3)	0.032 (3)	0.035 (3)	0.014 (2)	0.015 (3)	0.011 (2)
Cl1	0.1389 (16)	0.1335 (16)	0.1363 (16)	0.0487 (10)	0.0470 (10)	0.0583 (10)
Cl1'	0.1389 (16)	0.1335 (16)	0.1363 (16)	0.0487 (10)	0.0470 (10)	0.0583 (10)
C27	0.086 (6)	0.085 (6)	0.085 (6)	0.033 (2)	0.030 (2)	0.035 (2)

### Geometric parameters (Å, °)

**Table:**

Au—P1	2.2562 (15)	C9—H9C	0.9700
Au—S1	2.3029 (16)	C10—C11	1.429 (7)
Fe—C10	2.030 (5)	C10—C14	1.435 (7)
Fe—C10^i^	2.030 (5)	C11—C12	1.437 (8)
Fe—C14^i^	2.043 (5)	C11—H11	0.9400
Fe—C14	2.043 (5)	C12—C13	1.405 (9)
Fe—C11^i^	2.046 (5)	C12—H12	0.9400
Fe—C11	2.046 (5)	C13—C14	1.406 (8)
Fe—C13^i^	2.054 (6)	C13—H13	0.9400
Fe—C13	2.054 (6)	C14—H14	0.9400
Fe—C12	2.068 (5)	C15—C16	1.387 (8)
Fe—C12^i^	2.068 (5)	C15—C20	1.395 (7)
S1—C1	1.755 (6)	C16—C17	1.396 (8)
P1—C10	1.788 (5)	C16—H16	0.9400
P1—C21	1.817 (5)	C17—C18	1.391 (9)
P1—C15	1.824 (5)	C17—H17	0.9400
O1—C1	1.362 (7)	C18—C19	1.357 (10)
O1—C8	1.449 (7)	C18—H18	0.9400
N1—C1	1.277 (7)	C19—C20	1.399 (8)
N1—C2	1.398 (7)	C19—H19	0.9400
C2—C7	1.368 (9)	C20—H20	0.9400
C2—C3	1.398 (9)	C21—C22	1.387 (8)
C3—C4	1.384 (9)	C21—C26	1.393 (8)
C3—H3	0.9400	C22—C23	1.382 (8)
C4—C5	1.360 (10)	C22—H22	0.9400
C4—H4	0.9400	C23—C24	1.383 (9)
C5—C6	1.397 (10)	C23—H23	0.9400
C5—H5	0.9400	C24—C25	1.376 (10)
C6—C7	1.393 (9)	C24—H24	0.9400
C6—H6	0.9400	C25—C26	1.372 (9)
C7—H7	0.9400	C25—H25	0.9400
C8—C9	1.480 (9)	C26—H26	0.9400
C8—H8A	0.9800	Cl1—C27	1.701 (5)
C8—H8B	0.9800	Cl1—C27^ii^	1.74 (2)
C9—H9A	0.9700	C27—H27A	0.9800
C9—H9B	0.9700	C27—H27B	0.9800
			
P1—Au—S1	175.35 (5)	C9—C8—H8B	110.1
C10—Fe—C10^i^	180.0 (3)	H8A—C8—H8B	108.4
C10—Fe—C14^i^	138.7 (2)	C8—C9—H9A	109.5
C10^i^—Fe—C14^i^	41.3 (2)	C8—C9—H9B	109.5
C10—Fe—C14	41.3 (2)	H9A—C9—H9B	109.5
C10^i^—Fe—C14	138.7 (2)	C8—C9—H9C	109.5
C14^i^—Fe—C14	180.0 (3)	H9A—C9—H9C	109.5
C10—Fe—C11^i^	139.0 (2)	H9B—C9—H9C	109.5
C10^i^—Fe—C11^i^	41.0 (2)	C11—C10—C14	108.0 (5)
C14^i^—Fe—C11^i^	69.0 (2)	C11—C10—P1	122.9 (4)
C14—Fe—C11^i^	111.0 (2)	C14—C10—P1	129.0 (4)
C10—Fe—C11	41.0 (2)	C11—C10—Fe	70.1 (3)
C10^i^—Fe—C11	139.0 (2)	C14—C10—Fe	69.9 (3)
C14^i^—Fe—C11	111.0 (2)	P1—C10—Fe	127.3 (3)
C14—Fe—C11	69.0 (2)	C10—C11—C12	107.0 (5)
C11^i^—Fe—C11	180.0 (3)	C10—C11—Fe	68.9 (3)
C10—Fe—C13^i^	111.7 (2)	C12—C11—Fe	70.4 (3)
C10^i^—Fe—C13^i^	68.3 (2)	C10—C11—H11	126.5
C14^i^—Fe—C13^i^	40.1 (2)	C12—C11—H11	126.5
C14—Fe—C13^i^	139.9 (2)	Fe—C11—H11	125.8
C11^i^—Fe—C13^i^	68.3 (2)	C13—C12—C11	108.1 (5)
C11—Fe—C13^i^	111.7 (2)	C13—C12—Fe	69.5 (3)
C10—Fe—C13	68.3 (2)	C11—C12—Fe	68.7 (3)
C10^i^—Fe—C13	111.7 (2)	C13—C12—H12	126.0
C14^i^—Fe—C13	139.9 (2)	C11—C12—H12	126.0
C14—Fe—C13	40.1 (2)	Fe—C12—H12	127.4
C11^i^—Fe—C13	111.7 (2)	C14—C13—C12	109.3 (5)
C11—Fe—C13	68.3 (2)	C14—C13—Fe	69.5 (3)
C13^i^—Fe—C13	180.0 (3)	C12—C13—Fe	70.6 (4)
C10—Fe—C12	68.4 (2)	C14—C13—H13	125.3
C10^i^—Fe—C12	111.6 (2)	C12—C13—H13	125.3
C14^i^—Fe—C12	112.2 (2)	Fe—C13—H13	126.1
C14—Fe—C12	67.8 (2)	C13—C14—C10	107.5 (5)
C11^i^—Fe—C12	139.1 (2)	C13—C14—Fe	70.3 (3)
C11—Fe—C12	40.9 (2)	C10—C14—Fe	68.9 (3)
C13^i^—Fe—C12	140.1 (3)	C13—C14—H14	126.2
C13—Fe—C12	39.9 (3)	C10—C14—H14	126.2
C10—Fe—C12^i^	111.6 (2)	Fe—C14—H14	126.1
C10^i^—Fe—C12^i^	68.4 (2)	C16—C15—C20	120.0 (5)
C14^i^—Fe—C12^i^	67.8 (2)	C16—C15—P1	122.6 (4)
C14—Fe—C12^i^	112.2 (2)	C20—C15—P1	117.3 (4)
C11^i^—Fe—C12^i^	40.9 (2)	C15—C16—C17	119.3 (5)
C11—Fe—C12^i^	139.1 (2)	C15—C16—H16	120.3
C13^i^—Fe—C12^i^	39.9 (3)	C17—C16—H16	120.3
C13—Fe—C12^i^	140.1 (3)	C18—C17—C16	120.4 (6)
C12—Fe—C12^i^	180.0 (4)	C18—C17—H17	119.8
C1—S1—Au	102.6 (2)	C16—C17—H17	119.8
C10—P1—C21	106.8 (2)	C19—C18—C17	120.1 (5)
C10—P1—C15	105.4 (2)	C19—C18—H18	119.9
C21—P1—C15	103.4 (2)	C17—C18—H18	119.9
C10—P1—Au	115.79 (18)	C18—C19—C20	120.7 (6)
C21—P1—Au	112.8 (2)	C18—C19—H19	119.7
C15—P1—Au	111.77 (18)	C20—C19—H19	119.7
C1—O1—C8	116.2 (5)	C15—C20—C19	119.5 (6)
C1—N1—C2	121.6 (5)	C15—C20—H20	120.3
N1—C1—O1	120.3 (5)	C19—C20—H20	120.3
N1—C1—S1	126.6 (5)	C22—C21—C26	119.1 (5)
O1—C1—S1	113.1 (4)	C22—C21—P1	121.0 (4)
C7—C2—N1	119.6 (6)	C26—C21—P1	119.8 (5)
C7—C2—C3	118.6 (6)	C23—C22—C21	119.7 (6)
N1—C2—C3	121.5 (6)	C23—C22—H22	120.1
C4—C3—C2	119.6 (6)	C21—C22—H22	120.1
C4—C3—H3	120.2	C22—C23—C24	120.7 (6)
C2—C3—H3	120.2	C22—C23—H23	119.6
C5—C4—C3	121.4 (6)	C24—C23—H23	119.6
C5—C4—H4	119.3	C25—C24—C23	119.5 (6)
C3—C4—H4	119.3	C25—C24—H24	120.2
C4—C5—C6	119.9 (6)	C23—C24—H24	120.2
C4—C5—H5	120.1	C26—C25—C24	120.3 (6)
C6—C5—H5	120.1	C26—C25—H25	119.8
C7—C6—C5	118.4 (6)	C24—C25—H25	119.8
C7—C6—H6	120.8	C25—C26—C21	120.6 (6)
C5—C6—H6	120.8	C25—C26—H26	119.7
C2—C7—C6	122.1 (6)	C21—C26—H26	119.7
C2—C7—H7	119.0	Cl1^ii^—C27—Cl1	116.5 (11)
C6—C7—H7	119.0	Cl1—C27—H27A	108.2
O1—C8—C9	108.1 (5)	Cl1^ii^—C27—H27A	108.2
O1—C8—H8A	110.1	Cl1—C27—H27B	108.2
C9—C8—H8A	110.1	Cl1^ii^—C27—H27B	108.2
O1—C8—H8B	110.1	H27A—C27—H27B	107.3
			
P1—Au—S1—C1	106.4 (6)	C11^i^—Fe—C12—C13	59.9 (5)
S1—Au—P1—C10	160.4 (6)	C11—Fe—C12—C13	−120.1 (5)
S1—Au—P1—C21	−76.2 (6)	C13^i^—Fe—C12—C13	180.000 (1)
S1—Au—P1—C15	39.7 (6)	C12^i^—Fe—C12—C13	−49 (34)
C2—N1—C1—O1	−177.6 (5)	C10—Fe—C12—C11	38.5 (3)
C2—N1—C1—S1	1.0 (9)	C10^i^—Fe—C12—C11	−141.5 (3)
C8—O1—C1—N1	−1.7 (8)	C14^i^—Fe—C12—C11	−96.8 (3)
C8—O1—C1—S1	179.5 (5)	C14—Fe—C12—C11	83.2 (3)
Au—S1—C1—N1	−149.8 (5)	C11^i^—Fe—C12—C11	180.0
Au—S1—C1—O1	28.9 (5)	C13^i^—Fe—C12—C11	−59.9 (5)
C1—N1—C2—C7	115.8 (7)	C13—Fe—C12—C11	120.1 (5)
C1—N1—C2—C3	−70.2 (8)	C12^i^—Fe—C12—C11	71 (32)
C7—C2—C3—C4	−1.5 (9)	C11—C12—C13—C14	0.9 (6)
N1—C2—C3—C4	−175.5 (6)	Fe—C12—C13—C14	58.9 (4)
C2—C3—C4—C5	0.0 (10)	C11—C12—C13—Fe	−58.0 (4)
C3—C4—C5—C6	1.1 (10)	C10—Fe—C13—C14	−38.5 (3)
C4—C5—C6—C7	−0.6 (10)	C10^i^—Fe—C13—C14	141.5 (3)
N1—C2—C7—C6	176.1 (6)	C14^i^—Fe—C13—C14	180.0
C3—C2—C7—C6	2.0 (9)	C11^i^—Fe—C13—C14	97.2 (4)
C5—C6—C7—C2	−1.0 (10)	C11—Fe—C13—C14	−82.8 (4)
C1—O1—C8—C9	173.2 (6)	C13^i^—Fe—C13—C14	54 (58)
C21—P1—C10—C11	−160.4 (4)	C12—Fe—C13—C14	−120.4 (5)
C15—P1—C10—C11	90.1 (5)	C12^i^—Fe—C13—C14	59.6 (5)
Au—P1—C10—C11	−33.9 (5)	C10—Fe—C13—C12	81.9 (3)
C21—P1—C10—C14	22.0 (6)	C10^i^—Fe—C13—C12	−98.1 (3)
C15—P1—C10—C14	−87.4 (5)	C14^i^—Fe—C13—C12	−59.6 (5)
Au—P1—C10—C14	148.5 (4)	C14—Fe—C13—C12	120.4 (5)
C21—P1—C10—Fe	−71.4 (4)	C11^i^—Fe—C13—C12	−142.4 (3)
C15—P1—C10—Fe	179.2 (3)	C11—Fe—C13—C12	37.6 (3)
Au—P1—C10—Fe	55.1 (4)	C13^i^—Fe—C13—C12	174 (58)
C10^i^—Fe—C10—C11	−160 (45)	C12^i^—Fe—C13—C12	180.000 (1)
C14^i^—Fe—C10—C11	61.1 (5)	C12—C13—C14—C10	−0.5 (6)
C14—Fe—C10—C11	−118.9 (5)	Fe—C13—C14—C10	59.1 (4)
C11^i^—Fe—C10—C11	180.0	C12—C13—C14—Fe	−59.6 (4)
C13^i^—Fe—C10—C11	98.6 (4)	C11—C10—C14—C13	−0.1 (6)
C13—Fe—C10—C11	−81.4 (4)	P1—C10—C14—C13	177.8 (4)
C12—Fe—C10—C11	−38.4 (4)	Fe—C10—C14—C13	−60.0 (4)
C12^i^—Fe—C10—C11	141.6 (4)	C11—C10—C14—Fe	59.9 (4)
C10^i^—Fe—C10—C14	−41 (45)	P1—C10—C14—Fe	−122.2 (4)
C14^i^—Fe—C10—C14	180.0	C10—Fe—C14—C13	118.7 (5)
C11^i^—Fe—C10—C14	−61.1 (5)	C10^i^—Fe—C14—C13	−61.3 (5)
C11—Fe—C10—C14	118.9 (5)	C14^i^—Fe—C14—C13	−135 (100)
C13^i^—Fe—C10—C14	−142.5 (3)	C11^i^—Fe—C14—C13	−99.3 (4)
C13—Fe—C10—C14	37.5 (3)	C11—Fe—C14—C13	80.7 (4)
C12—Fe—C10—C14	80.5 (4)	C13^i^—Fe—C14—C13	180.0
C12^i^—Fe—C10—C14	−99.5 (4)	C12—Fe—C14—C13	36.7 (4)
C10^i^—Fe—C10—P1	83 (45)	C12^i^—Fe—C14—C13	−143.3 (4)
C14^i^—Fe—C10—P1	−55.7 (5)	C10^i^—Fe—C14—C10	180.0
C14—Fe—C10—P1	124.3 (5)	C14^i^—Fe—C14—C10	106 (100)
C11^i^—Fe—C10—P1	63.2 (5)	C11^i^—Fe—C14—C10	142.0 (3)
C11—Fe—C10—P1	−116.8 (5)	C11—Fe—C14—C10	−38.0 (3)
C13^i^—Fe—C10—P1	−18.3 (4)	C13^i^—Fe—C14—C10	61.3 (5)
C13—Fe—C10—P1	161.7 (4)	C13—Fe—C14—C10	−118.7 (5)
C12—Fe—C10—P1	−155.2 (4)	C12—Fe—C14—C10	−82.1 (3)
C12^i^—Fe—C10—P1	24.8 (4)	C12^i^—Fe—C14—C10	97.9 (3)
C14—C10—C11—C12	0.6 (6)	C10—P1—C15—C16	−2.2 (5)
P1—C10—C11—C12	−177.4 (4)	C21—P1—C15—C16	−114.1 (5)
Fe—C10—C11—C12	60.4 (4)	Au—P1—C15—C16	124.4 (4)
C14—C10—C11—Fe	−59.8 (4)	C10—P1—C15—C20	−178.4 (4)
P1—C10—C11—Fe	122.2 (4)	C21—P1—C15—C20	69.7 (5)
C10^i^—Fe—C11—C10	180.0	Au—P1—C15—C20	−51.9 (5)
C14^i^—Fe—C11—C10	−141.8 (3)	C20—C15—C16—C17	−1.2 (8)
C14—Fe—C11—C10	38.2 (3)	P1—C15—C16—C17	−177.3 (4)
C11^i^—Fe—C11—C10	8(100)	C15—C16—C17—C18	−0.1 (9)
C13^i^—Fe—C11—C10	−98.6 (4)	C16—C17—C18—C19	0.8 (9)
C13—Fe—C11—C10	81.4 (4)	C17—C18—C19—C20	−0.3 (10)
C12—Fe—C11—C10	118.1 (5)	C16—C15—C20—C19	1.7 (8)
C12^i^—Fe—C11—C10	−61.9 (5)	P1—C15—C20—C19	178.1 (4)
C10—Fe—C11—C12	−118.1 (5)	C18—C19—C20—C15	−0.9 (9)
C10^i^—Fe—C11—C12	61.9 (5)	C10—P1—C21—C22	−73.8 (5)
C14^i^—Fe—C11—C12	100.1 (4)	C15—P1—C21—C22	37.1 (5)
C14—Fe—C11—C12	−79.9 (4)	Au—P1—C21—C22	157.9 (4)
C11^i^—Fe—C11—C12	−110 (100)	C10—P1—C21—C26	108.5 (5)
C13^i^—Fe—C11—C12	143.3 (4)	C15—P1—C21—C26	−140.7 (4)
C13—Fe—C11—C12	−36.7 (4)	Au—P1—C21—C26	−19.8 (5)
C12^i^—Fe—C11—C12	180.0	C26—C21—C22—C23	0.2 (8)
C10—C11—C12—C13	−0.9 (6)	P1—C21—C22—C23	−177.6 (4)
Fe—C11—C12—C13	58.5 (4)	C21—C22—C23—C24	0.6 (9)
C10—C11—C12—Fe	−59.4 (4)	C22—C23—C24—C25	−1.4 (9)
C10—Fe—C12—C13	−81.5 (4)	C23—C24—C25—C26	1.5 (10)
C10^i^—Fe—C12—C13	98.5 (4)	C24—C25—C26—C21	−0.7 (10)
C14^i^—Fe—C12—C13	143.1 (3)	C22—C21—C26—C25	−0.1 (9)
C14—Fe—C12—C13	−36.9 (3)	P1—C21—C26—C25	177.7 (5)

Symmetry codes: (i) −*x*, −*y*, −*z*; (ii) −*x*, −*y*+1, −*z*+1.

### Hydrogen-bond geometry (Å, °)

**Table:**

Cg1 and Cg2 are the centroids of the C2–C7 and C15–C20 rings, respectively.

**Table:**

*D*—H···*A*	*D*—H	H···*A*	*D*···*A*	*D*—H···*A*
C9—H9a···Cg1^iii^	0.97	2.75	3.623 (9)	150
C11—H11···Cg2^iv^	0.94	2.78	3.619 (7)	150

Symmetry codes: (iii) *x*−1, *y*, *z*; (iv) −*x*+1, −*y*, −*z*.
